# Transmission Dynamics of COVID-19 in Ghana and the Impact of Public Health Interventions

**DOI:** 10.4269/ajtmh.21-0718

**Published:** 2022-05-23

**Authors:** Sylvia K. Ofori, Jessica S. Schwind, Kelly L. Sullivan, Benjamin J. Cowling, Gerardo Chowell, Isaac Chun-Hai Fung

**Affiliations:** ^1^ Department of Biostatistics, Epidemiology and Environmental Health Sciences, Jiann-Ping Hsu College of Public Health, Georgia Southern University, Statesboro, Georgia;; ^2^ WHO Collaborating Centre for Infectious Disease Epidemiology and Control, School of Public Health, Li Ka Shing Faculty of Medicine, The University of Hong Kong, Hong Kong Special Administrative Region;; ^3^ Department of Population Health Sciences, School of Public Health, Georgia State University, Atlanta, Georgia

## Abstract

This study characterized COVID-19 transmission in Ghana in 2020 and 2021 by estimating the time-varying reproduction number (*R_t_*) and exploring its association with various public health interventions at the national and regional levels. Ghana experienced four pandemic waves, with epidemic peaks in July 2020 and January, August, and December 2021. The epidemic peak was the highest nationwide in December 2021 with *R_t_* ≥ 2. Throughout 2020 and 2021, per-capita cumulative case count by region increased with population size. Mobility data suggested a negative correlation between *R_t_* and staying home during the first 90 days of the pandemic. The relaxation of movement restrictions and religious gatherings was not associated with increased *R_t_* in the regions with fewer case burdens. *R_t_* decreased from > 1 when schools reopened in January 2021 to < 1 after vaccination rollout in March 2021. Findings indicated most public health interventions were associated with *R_t_* reduction at the national and regional levels.

As of January 15, 2022, 153,514 cases of COVID-19 were confirmed in Ghana, with 1,343 deaths and 9,020 active cases.^1^ Among African countries, Ghana has controlled COVID-19 transmission with a substantial package of public health and social measures (Oxford stringency index of 62.04 as of May 7, 2020),^2^^,^^3^ including a 14-day mandatory quarantine for all persons who entered the country, school and church closures, a lockdown of major cities, and internal movement restrictions.^4^

This study describes the COVID-19 transmission dynamics in Ghana by estimating the time-varying reproduction number (*R_t_*) using the method of Cori et al.^5^ and a generalized growth model (Supplemental Information)^6^ to assess the impact of interventions at the national and regional levels and to explore the association between population size and the COVID-19 cumulative incidence. The correlations between population mobility and incident case count, and between mobility and *R_t_* were also assessed.

The daily number of new infections and daily cumulative incidence data by date of report for Ghana and each of its 16 regions was obtained through the Johns Hopkins University COVID-19 dashboard^7^ from March 12, 2020 to December 31, 2021 (Supplemental Figures S1 and S2, and Supplemental Table S1). To account for testing delay (3 days) and incubation period (6 days), the time series was shifted by 9 days to approximate the date of infection.^8^ Using 3-day moving averages of interpolated daily incident case count data, we used the EpiEstim package in R version 4.0.3 (R Foundation for Statistical Computing, Vienna, Austria) to estimate *R_t_* using the 7-day sliding window and the nonoverlapping time window between interventions.^5^ To compare *R_t_* before, during, and after policies were implemented and assess their association with COVID-19 transmission, specific time points at which a bundle of interventions began were selected for the latter analysis, and the average *R_t_* estimates over the period between two policy change time points were estimated (Table 1^1^^,9–11^).

**Table 1:** Public health and social measures interventions against COVID-19 implemented in Ghana

Date	Label assigned	Intervention	Location
March 15, 2020	A	Restricting all air travel to Ghana Suspension of all public gatherings, including workshops, funerals, and religious activities	National
March 16, 2020		Closure of all universities, senior high schools, and basic schools	National
March 17, 2020		Mandatory 14-day quarantine for all travelers entering the country Travel bans for all travelers who are not Ghanaian citizens or hold resident permits from countries with at least 200 cases Isolation and testing of symptomatic individuals Activation of contact tracing	National
March 22, 2020	B	Closure of all borders to human traffic for 2 weeks and suspension of passport services	National
March 26, 2020		Recall of staff on study leave by the Director-General of the Ghana Health Service	National
March 28, 2020		Offer of special health insurance coverage offered to all frontline workers	National
March 30, 2020	L	Lockdown of major cities in two regions	Regional
April 3, 2020		Announcement by the Director-General of the Ghana Health Service that nose masks are going to be manufactured locally Disinfection of markets in the northern, northeastern, Savannah, and eastern regions	Regional
April 5, 2020		Extension of border closure for another 2 weeks	National
April 27, 2020	C	Mandatory wearing of a mask at all businesses and organizations	National
May 11, 2020		Permission to operate of hotels, bars, and restaurants if following social distancing measures Extension of the ban of social gathering until the end of May	National
June 5, 2020	D	Relaxation of restrictions at social gatherings, including church services, with mandatory mask-wearing and maximum attendance of 100	National
June 15, 2020		Presidential announcement of a fine for those who refuse to wear a mask in public places Return to school by final-year students in tertiary institutions	National
June 21, 2020		Presidential approval of the construction of hospital-related facilities in the Greater Accra and Ashanti regions	Regional
July 6, 2020	E	Deployment of personnel to monitor COVID-19 cases in high schools	National
July 18, 2020		Two-phase fumigation exercise	National
July 26, 2020		COVID-19-related restrictions on transport operators and tourist sites lifted	National
August 1, 2020		Ease of restrictions on religious activities; duration of service increases from 1 hour to 2 hours	National
September 1, 2020	F	Reopening of international air border with mandatory testing of all passengers	National
September 7, 2020		Announcement of relief package for private schools affected by school closures	National
January 9, 2021	G	Re-opening of schools	National
March 1, 2021	V	Vaccine rollout	National
December 15, 2021	H	Behavioral changes related to Christmas festivities	National

The power-law relationship between cumulative case number and population size was investigated at 10 time points in 2020 through 2021 using linear regression of log_10_-transformed per-capita cumulative case number and log_10_-transformed population size.^12^ The relationship between the 7-day moving average of mobility changes and the 3-day moving average of daily number of new infections, and that between the 7-day moving average of mobility changes and the 7-day sliding window *R_t_* in the first 90 days of the pandemic were assessed using the time-lagged cross-correlation with data from Google Mobility Report.^13^ The Georgia Southern University Institutional Review Board determined a non-human subject status for this project (H20364) under the G8 exemption category.

Ghana and its regions experienced four pandemic waves in 2020 and 2021, with epidemic peaks in July 2020 and January, August, and December 2021. At the national level and in the Greater Accra and Ashanti regions, *R_t_* fluctuated around 1, increased to > 1 before the epidemic peaks, and dropped to < 1 afterward (Figure 1). The epidemic peak was the highest nationwide and in the Greater Accra region in December 2021, with an estimated *R_t_* of ≥ 2. Ghana’s December 2021 epidemic peak was largely driven by the Greater Accra region, whereas in other regions, case counts were increasing fast. Similar patterns in epidemic curves and *R_t_* estimates were observed in the central, eastern, Volta, and western regions, with *R_t_* ≥ 3 in December 2021 (Supplemental Figure S3). Meanwhile, using the generalized growth model, the COVID-19 *R_t_* estimate for Ghana was estimated at 1.8 (95% CI: 1.7, 2) for the first 15 days of the epidemic (Supplemental Figure S4).

**Figure 1.**
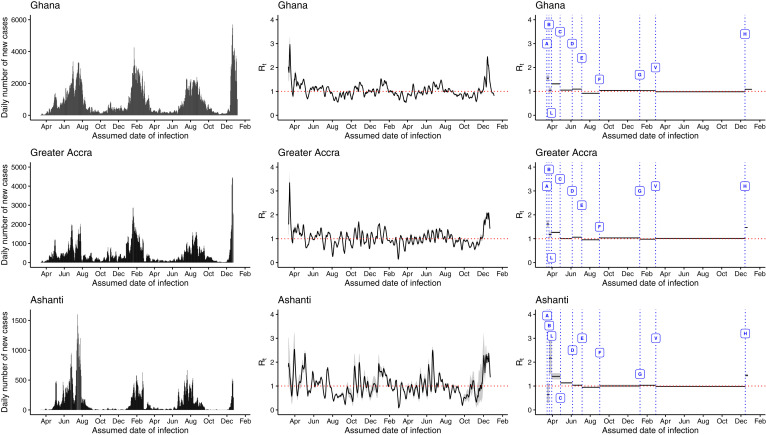
The daily number of new cases (**left panels**), 7-day sliding window time-varying reproduction number (*R_t_*; **middle panels**), and nonoverlapping window *R_t_* (**right panels**) estimated using the in the EpiEstim package for the Ghana, Greater Accra, and Ashanti regions, March 12, 2020 to December 31, 2021. The government policies represented by the alphabets in the figure are A = restriction of all air travel to Ghana, suspension of social gatherings, school closure, mandatory 14-day quarantine for all travelers; B = closure of all borders to human traffic; L= lockdown of major cities; C = mandatory wearing of a mask at all businesses and organizations; D = relaxation of restrictions at social gatherings; E = deployment of personnel to monitor COVID-19 cases in high schools; F = reopening of international borders; G = re-opening of schools; V = vaccination rollout, H= Christmas festivities in 2021. The Greater Accra and Ashanti regions are highlighted because they are the most populous and had the highest case burden in Ghana. This figure appears in color at www.ajtmh.org.

The *R_t_* estimates obtained using nonoverlapping windows suggested varying associations between government policies and interventions, and the increase and decrease of COVID-19 transmission in Ghana and across regions. The restriction of social gatherings and travel bans implemented on March 15, 2020 were associated with insignificant changes in *R_t_* at the national level and the Greater Accra region. However, when all borders were closed to human traffic nationwide a week later, *R_t_* decreased to around 1, accounting for a 32.63% (95% credible interval [CrI], 22.87–41.19) decrease for Ghana and a 27.41% (95% CrI, 16.68–36.26) decrease in the Greater Accra region. In contrast, *R_t_* for Ashanti increased by more than 100%. On April 27, 2020, the mandatory wearing of masks at all businesses and organizations was implemented nationwide, and was associated with a decrease in *R_t_* by 19.97% (95% CrI, 18.05–21.81) at the national level. The Ashanti and Greater Accra regions also observed significant declines in *R_t_*. The relaxation of restrictions on social gatherings was associated with a slight increase in *R_t_* by 4.03% (95% CrI, 2.61–5.49) for Ghana at the national level and by 6.02% (95% CrI, 4.05–7.78) in the Greater Accra region; however, the other regions observed a decline in *R_t_* by > 5%. Reopening of schools in January 2021 was associated with an increase in transmission in the eastern and central regions only. Vaccination rollout was associated with a decline in *R_t_* to < 1 in Ghana and the regions except the Greater Accra region, which observed about a 3% increase. Overall, behavioral changes resulting from Christmas festivities in 2021 were associated with sustained transmission in all regions. Details of percentage changes in *R_t_* are included in Supplemental Table S2.

The assessment of population size and cumulative incidence showed that Ghanaian regions with larger populations experienced higher COVID-19 attack rates (Figure 2 and Supplemental Table S3). Furthermore, there was a weak correlation between mobility changes and COVID-19 incidence in the first 90 days of the pandemic in Ghana (Supplemental Tables S4 and S5). For example, the daily number of new infections correlated positively with mobility changes to retail and recreation facilities, grocery and pharmacy facilities, and workplaces 3 days later (*r *> 0, *P* < 0.05 in all three cases), but no significant correlation was observed with residential mobility changes. *R_t_* correlated negatively with mobility changes to residences (*r *= –0.328, *P* = 0.002).

**Figure 2.**
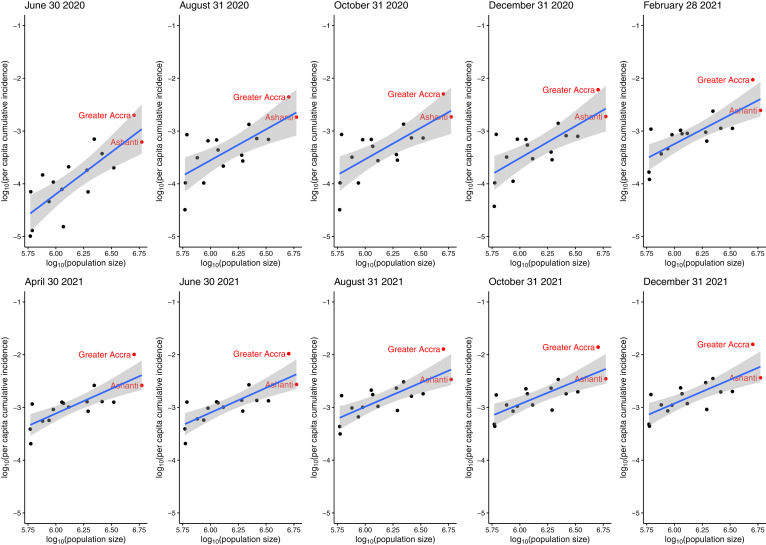
Linear regression models between log_10_-transformed per-capita cumulative case count and log_10_-transformed population size of the 16 regions of Ghana (gray areas represent the 95% CIs of the regression lines) at 10 time points: June 30, August 31, October 31, and December 31, 2020; and February 28, April 30, June 30, August 31, October 31, and December 31, 2021. This figure appears in color at www.ajtmh.org.

Overall, *R_t_* > 1 showed sustained transmission across the country, but some interventions implemented during the study period were associated with reduced *R_t_* in regions with relatively higher case counts. Interestingly, relaxation of restrictions at social gatherings was not associated with an increase in *R_t_*.

The early estimates of *R_t_* for Ghana were less than the reported values for other countries such as Nigeria,^14^ Egypt,^15^ and Kenya.^16^ The lower *R* values for Ghana suggest that public health and social measures were effective in containing the epidemic at the initial stage. The estimates differ slightly from those of Dwomoh et al.,^17^ but may be explained by the differences in methods, including the fact that they used data from the first 60 days. Nevertheless, *R_t_* remained > 1 at the national level and in most regions, indicating sustained transmission. It is therefore imperative that public health measures be strengthened throughout the country, and efforts be prioritized, especially in regions with larger population sizes, because the disparity in the case burden across regions was reported in multiple studies.^18^^,^^19^ This disparity may be explained by the difficulty in practicing social distancing because of overcrowding stemming from high commercial activities, slum areas facilitating disease spread, and urban residency. Hence, such regions will be required to implement more stringent preventive measures to decrease transmission.^20^

The reopening of schools was associated with a surge in cases in other jurisdictions.^21^ This finding supports the need for routine surveillance, case investigation, better protocols for isolation and quarantine, and deployment of protective personal equipment to schools. Although the correlation between changes in mobility and transmission intensity was weak in our study, a decline in trips to grocery and pharmacy outlets and workplaces was reported to be associated with lower transmission rates in most countries before and after interventions were relaxed.^22^ The difference in correlation between mobility changes and transmission intensity may be due to cultural differences, economic status, and variations in the types of interventions implemented.

It was unexpected that the relaxation of restrictions on social gatherings was not associated with increase in *R_t_* estimates, although such policies make it difficult to practice social distancing, especially in enclosed places such as churches or restaurants. This finding may be observed as a result of the residual effect of the prior mask mandates and the reluctance of prominent churches to resume in-person religious activities.^23^ In addition, mobility changes to retail stores and recreation centers remained below baseline even after the restrictions were relaxed (Supplemental Figure S5).

Our study is not without limitations. First, we used publicly available data, which were subject to underreporting or reporting delays. Second, data were only available by the date of the report and unavailable by the date of symptom onset. Therefore, we accounted for this situation by shifting the data by 9 days to approximate the time of infection. It is possible that this method of approximating the date of symptom affected the results of the cross-correlation analysis, which suggested that changes in the daily number of new infections correlated with mobility changes 3 days later. Third, given the use of aggregate data, individual-level assumptions cannot be made. Fourth, we cannot rule out the possibility of ceiling effects during months when testing capacity was limited (Supplemental Figure S6). Last, socioeconomic data were limited at the regional level; hence, further exploration of the case burden by region could not be performed.

In conclusion, most of the interventions implemented by the Ghanaian government were followed by an overall decrease in *R_t_* estimates at the national scale, but a decline was not seen across all regions. Our results highlight the importance of a sustained, multi-faceted response at the national level to help mitigate the varying regional effects observed during this pandemic.

## Supplemental Material


Supplemental materials
