# Supplementary material for: Transmission Dynamics of COVID-19 in Ghana and the Impact of Public Health Interventions
Source: Am J Trop Med Hyg. 2022 May 23;107(1):175–9. doi: 10.4269/ajtmh.21-0718 (PMC9294683; doi:10.4269/ajtmh.21-0718)
Supplement: Supplementary file 1 [file tpmd210718.SD1.pdf]

Supplemental Materials

Supplementary text for Methods

Data interpolation and moving averages

The data from obtained from the Johns Hopkins University was interpolated to estimate the values of missing data. The interpolated values were used to estimate the 3-day moving averages to reduce fluctuations in analysis.

Timeline for regions

At the regional level, the time series began when the first cases were reported consecutively and ended after cases were consecutively reported. For example, for Central Region, the time series began on 7th May 2020. This was done to avoid estimating the R_t too early in the pandemic and to minimize error and uncertainty around the R_t. Six out of the sixteen regions were included in the analysis based on having more than 4,000 cumulative cases as of December 31, 2021, to avoid the fluctuations in the estimates (see Figures S1 and S2).

The reproduction number, R_t, from daily incidence using the generalized growth model (GGM)

The daily number of new infections for the first 15 days of the epidemic and selected regional levels are calibrated using the Generalized Growth Model (GGM).[1] The GGM uses a simple generalized model for the ascending case of an epidemic using the equation:

$$\frac{dC(t)}{dt} = C'(t) = rC(t)^p$$

where $C'(t)$ characterizes the epidemic curve (incident case count) over the time t and the solution C(t) which is the cumulative incidence at a given time t, r is a positive number defined as the growth rate (1/t), and $p \in [0, 1]$ is the "deceleration of growth parameter". At a p of zero, the equation describes a constant incidence and at p=1, the equation outlines an exponential growth dynamics. Values of p between 0 and 1 describe sub-exponential growth patterns.[2]

We first assume a gamma distribution for the generation interval of SARS-CoV-2 with a mean of 4.60 days and a standard deviation of 5.55 days,[3, 4] then estimate the growth rate parameter r, and the deceleration of growth parameter, p. The GGM model simulates the growth of total cases (both imported and local cases) at I_i, and estimates the reproduction using the renewal equation:[5]

$$R_{t_i} = \frac{I_i}{\sum_{j=0}^{i}(I_{i-j} + \alpha J_{i-j})\rho_j}$$

The imported cases at a given time t are given as J_i; the local incidence is I_i at calendar time t_i; and ρ_j represents the discretized probability distribution of generation interval. The factor α measures the relative contribution of imported cases to the transmission process and is assumed to be 0.15. The total number of new cases is the numerator at a given time $t= I_i$, and the denominator is the total number of primary cases that generated the secondary cases. Thus, the reproduction number is the average number of new cases generated by a primary case at a given calendar time. The uncertainty around the R_t is derived from the parameter estimates (r, p). The R_t is estimated from 300 simulations assuming a negative binomial structure with a mean which is assumed to be a third of the variance.[2] The R_t for the national curve is estimated from the first fifteen days of infection (March 12th – March 26, 2020).

Time-varying reproduction number R_t, using the Instantaneous Reproduction Number method (Cori et al. method).

The R_t is used because it can be estimated easily in real-time and when control measures are implemented.

The instantaneous reproduction number method in the R package EpiEstim developed by Cori et al and Thompson et al. was used in the analysis.[6, 7] The EpiEstim package uses a Bayesian framework assuming a gamma prior distribution for the posterior distribution for R_t.[3, 6, 8] The parametric method was used in which the serial interval was assumed to follow a gamma distribution with a mean of 4.60 days and a standard deviation of 5.55.[3, 4]

R_t using 7-day sliding windows.

The instantaneous reproduction number was estimated over a 7-day sliding window given the high variation in daily R_t estimates. The R_t was assumed to be constant in each time window and the average of R_t estimates over 7 days was estimated with its credible intervals.

R_t using non-overlapping windows to assess the impact of various interventions.

This method is used to compare the R_t before, during, and after a policy is implemented to assess its impact on transmission.[7] An average was taken of the R_t estimates over the period between two policy change time points. The data on the major interventions and newly implemented policies were obtained from the Ghana Health Service website as well as the websites of the major news media. The following data were collected: travel bans and border closures, restriction of social activities, school closure, the mandatory wearing of masks, relaxation of restrictions on social and religious activities, deployment of personnel to monitor COVID-19 in senior high schools, reopening of air borders, and reopening of schools. A detailed list of

interventions and their references can be found in Table 1. Bundled policies were implemented in close succession; hence there is difficulty in assessing the individual effects. **Regression analysis**

The power-law relationship between cumulative case count, C, and population size, N (i.e., $C \sim N^g$ where g is an exponent) was transformed into a relationship between $\log_{10}$-transformed per-capita cumulative case count and $\log_{10}$-transformed population size as follows:[9]

$$\frac{\log_{10}(\text{cumulative case count})}{\log_{10}(\text{population size})} = g$$

$$\frac{\log_{10}(\text{per capita cumulative case count} \times \text{population size})}{\log_{10}(\text{population size})} = g$$

$$\frac{\log_{10}(\text{per capita cumulative case count})}{\log_{10}(\text{population size})} + \frac{\log_{10}(\text{population size})}{\log_{10}(\text{population size})} = g$$

$$\frac{\log_{10}(\text{per capita cumulative case count})}{\log_{10}(\text{population size})} = g - 1 = m$$

The per capita cumulative case count was calculated by dividing the total cumulative case count by the population size of the nation or region at the specified time point. A linear regression model was fit to the data of $\log_{10}$-transformed per capita cumulative case count and $\log_{10}$-transformed population size. The slope m of the regression line (where $m=g$-1) can be interpreted as follows: if $m=0$, there is a homogeneity of per capita cumulative case count across regions; if $m<0$, low-population regions have higher per capita cumulative case count than high-population regions; and if $m>0$, low-population regions have lower per capita cumulative case count than

high-population regions.[9] The 2020 population estimates for Ghana and the regions were obtained from the Ghana Statistical Service.[10]

Google mobility data

The mobility data was sourced from Google to analyze changes in the number of visits to places in the following categories: (a) grocery and pharmacy, (b) parks, (c) transit stations, (d) retail and recreation, (e) residential, and (f) workplaces.[11] The data provides information for how visits and duration of staying at different places changes compared to baseline values which is the median value for the corresponding day of the week, during the five weeks of January 3 — February 6, 2020. The data is representative of users who opted for location history. The relationship between the 7-day moving average of mobility changes in retails and recreation, grocery and pharmacy, and workplace, and the 3-day moving average of the daily number of new infections (or R_t estimates from a 7-day sliding window) is assessed using the time-lagged cross-correlation using the assumed dates of infection.

Supplementary text for Results

Epidemic curves

Four waves were observed in the epidemic curves for Ghana. The daily number of new cases of COVID-19 in Ghana, the Greater Accra Region, and the Ashanti region decreased from mid-May to mid-June and surged in mid-July, 2020. For all three locations, the daily number of new cases dropped again to less than 500 per day and spiked again in January 2021 (Figure 1). Relatively few new cases were observed between March and July 2021, before rising again in late July. Very high numbers of new cases were recorded from December 2021 due to Christmas festivities. The Western region’s daily new cases were highest in June and July 2020, and

January-February 2021 (Figure S3). The daily number of new cases for the Central, Volta, and Eastern regions fluctuated throughout the study period but followed a similar trajectory with that of Ghana as a whole in the peak periods (Figures S3).

Comparing R_t Estimates using GGM and Cori et al. method.

Using the GGM, the COVID-19 R_t estimate for Ghana was estimated at 1.8 (95% CI: 1.7, 2) for the first 15 days of the epidemic (Figure S4) compared to that estimated using the Cori et al. method, which estimated the R_t at 1.85 (95% CI: 1.42, 2.27).

The difference in estimates may be because in the Cori et al. method, fluctuations in daily number of new infections are treated as true reflections of the underlying epidemic while the GGM method treats these fluctuations as noise by assuming an error structure underneath. Hence, the estimated value of R_t using the Cori et al. method may provide a better reflection of how early interventions and policy changes affected the R_t at the national level for this study.

REFERENCES

1. Viboud C, Simonsen L, Chowell G. A generalized-growth model to characterize the early ascending phase of infectious disease outbreaks. *Epidemics*. 2016;15:27-37.
2. Chowell G. Fitting dynamic models to epidemic outbreaks with quantified uncertainty: A primer for parameter uncertainty, identifiability, and forecasts. *Infectious Disease Modelling*. 2017;2(3):379-398.
3. Fung IC-H, Hung YW, Ofori SK, Muniz-Rodriguez K, Lai P-Y, Chowell G. SARS-CoV-2 Transmission in Alberta, British Columbia, and Ontario, Canada, December 25, 2019, to December 1, 2020. *Disaster Medicine and Public Health Preparedness*. 2021:1-10. doi:doi:10.1017/dmp.2021.78
4. You C, Deng Y, Hu W, et al. Estimation of the time-varying reproduction number of COVID-19 outbreak in China. *International Journal of Hygiene Environmental Health*. 2020;228:113555.
5. Nishiura H, Chowell G. Early transmission dynamics of Ebola virus disease (EVD), West Africa, March to August 2014. *Eurosurveillance*. 2014;19(36):20894.
6. Cori A, Ferguson NM, Fraser C, Cauchemez S. A New Framework and Software to Estimate Time-Varying Reproduction Numbers During Epidemics. *American Journal of Epidemiology*. 2013;178(9):1505-1512. doi:10.1093/aje/kwt133
7. Thompson R, Stockwin J, van Gaalen RD, et al. Improved inference of time-varying reproduction numbers during infectious disease outbreaks. *Epidemics*. 2019;29:100356.
8. Fraser C, Cummings DA, Klinkenberg D, Burke DS, Ferguson NM. Influenza transmission in households during the 1918 pandemic. *American journal of epidemiology*. 2011;174(5):505-514.
9. Chowell G, Bettencourt LM, Johnson N, Alonso WJ, Viboud C. The 1918–1919 influenza pandemic in England and Wales: spatial patterns in transmissibility and mortality impact. *Proceedings of the Royal Society B: Biological Sciences*. 2008;275(1634):501-509.
10. Ghana Statistical Service. Ghana's population by region, 2020. Accessed 04/07/2021, 2021. https://www.statsghana.gov.gh/index.php?id=MjYzOTE0MjAuMzc2NQ==/webstats/4238n0op4p
11. Google LLC. Google COVID-19 Community Mobility Reports. Accessed April 27, 2021, https://www.google.com/covid19/mobility

Supplemental Table S1: Cumulative number of cases and cumulative incidence rate by region as of December 31, 2021 (N= 142,986)

Region	Population	Cumulative Number of Cases	Cumulative Number of Cases per 100,000
Ghana (national)	32,000,000	142,986	446.83
Ahafo	613,000	1,074	175.20
Ashanti	5,924,500	21,646	365.36
Bono	1,168,800	2,119	181.30
Bono East	1,133,800	2,667	235.23
Central	2,605,500	5,143	197.39
Eastern	3,318,900	6,661	200.70
Greater Accra	5,055,900	79,567	1573.75
North East	588,800	283	48.06
Northern	1,948,900	1,787	91.69
Oti	759,800	850	111.87
Savannah	594,700	263	44.22
Upper East	1,302,700	1,484	113.92
Upper West	868,500	1,543	177.66
Volta	1,907,700	5,621	294.65
Western	2,214,700	7,817	352.96
Western North	949,100	1,049	110.53

Supplemental Table S2: Median estimates and 95% credible intervals of nonoverlapping window R_t for each period between each policy change (left) and percent changes in nonoverlapping window R_t and associated 95% credible intervals with given policies.

	R_t		Percentage change (%)	
**Policy change**	**Median**	**95% Credible Interval**	**Median**	**95% Credible Interval**
**National**				
Before A	1.92	1.54, 2.34		
A to B	1.54	1.42, 1.67	-20.19	-34.75, +3.91
B to L	1.05	0.94, 1.16	-32.63	-41.19, -22.87
L to C	1.31	1.29, 1.34	+25.62	+13.60, +40.42
C to D	1.05	1.04, 1.06	-19.97	-21.81, -18.05
D to E	1.09	1.09, 1.10	+4.03	+2.61, +5.49
E to F	0.92	0.91, 0.93	-15.95	-16.86, -15.10
F to G	1.04	1.03, 1.05	+13.01	+11.74, +14.25
G to V	1.03	1.03, 1.04	-0.60	-1.58, 0.49
V to H	0.98	0.98, 0,99	-4.80	-5.51, -4.09
Beyond H	1.09	1.08, 1.10	+10.57	+9.62, +11.47
**Regions**				
**Greater Accra**				
Before A	1.75	1.35, 2.21		
A to B	1.62	1.47, 1.77	-7.48	-27.84, +20.48
B to L	1.17	1.06, 1.29	-27.41	-36.26, -16.68
L to C	1.27	1.23, 1.29	+7.70	-2.70, +19.31
C to D	1.00	0.99, 1.02	-20.84	-22.88, -18.75
D to E	1.06	1.05, 1.07	+6.02	+4.05, +7.78
E to F	0.95	0.94, 0.96	-10.07	-11.45, -8.68
F to G	1.03	1.02, 1.04	+8.24	+6.87, +9.64
G to V	0.98	0.97, 0.99	-5.30	-6.47, -4.30
V to H	1.01	1.00, 1.02	+3.43	+2.35, +4.46

Beyond H	1.47	1.45, 1.49	+45.15	+43.33, +46.99
**Ashanti**				
Before A	1.84	1.20, 2.69		
A to B	0.61	0.29, 1.11	-66.67	-85.65, -32.54
B to L	2.15	1.50, 2.96	+250.86	+84.23, +681.67
L to C	1.40	1.27, 1.54	-35.34	-52.66, -6.37
C to D	1.13	1.10, 1.16	-19.34	-26.19, -11.69
D to E	1.03	1.02 1.05	-8.49	-11.08, -5.82
E to F	0.95	0.93, 0.96	-8.43	-10.45, -6.53
F to G	1.00	0.95, 1.05	+5.75	+0.35, +10.98
G to V	1.03	1.01, 1.05	+2.87	-2.34, +8.52
V to H	0.98	0.97, 0.99	-4.84	-6.67, -2.27
Beyond H	1.45	1.39, 1.50	+47.31	+41.77, +52.84
**Central**				
Before C	1.47	1.04, 1.99		
C to D	1.16	1.10, 1.22	-20.86	-42.14, +14.15
D to E	1.06	1.00, 1.09	-9.65	-15.07, -3.46
E to F	0.94	0.91, 0.97	-9.99	-14.28, -5.25
F to G	0.94	0.88, 0.99	-0.58	-7.17, +6.52
G to V	1.16	1.12, 1.20	+23.31	+15.66, +32.75
V to H	0.95	0.93, 0.97	-17.86	-21.02, -14.39
Beyond H	1.24	1.00, 1.51	+29.70	+5.24, +59.34
**Eastern**				
Before C	1.21	1.09, 1.34		
C to D	1.21	1.11, 1.32	+0.63	-12.97, +15.60
D to E	1.05	1.01, 1.08	-13.89	-21.62, -4.77
E to F	0.99	0.96, 1.01	-5.54	-9.76, -1.18
F to G	0.88	0.84, 0.94	-10.15	-15.58, -4.52
G to V	1.05	1.02, 1.07	+17.86	+10.66, +26.23

V to H	0.98	0.96, 0.99	-6.73	-9.74, -3.58
Beyond H	1.55	1.35, 1.76	+58.30	+40.16, +80.74
**Volta**				
Before C	1.73	1.40, 2.12		
C to D	1.43	1.33, 1.54	-18.29	-31.95, +3.86
D to E	0.85	0.80, 0.91	-40.35	-45.64, -34.66
E to F	0.90	0.84, 0.96	+4.92	-4.71, +15.57
F to G	1.06	0.97, 1.16	+18.83	+5.92, +31.69
G to V	1.08	1.04, 1.12	+1.06	-7.43, +11.28
V to H	0.97	0.95, 0.99	-10.08	-12.68, -6.92
Beyond H	2.20	2.02, 2.39	+127.92	+109.66, +148.11
**Western**				
Before C	1.53	1.24, 1.87		
C to D	1.26	1.22, 1.30	-18.77	-33.71, +0.40
D to E	0.97	0.94, 0.99	-23.26	-26.55, -19.97
E to F	0.85	0.82, 0.88	-11.71	-15.88, -7.83
F to G	1.12	1.05, 1.19	+31.58	+22.22, +41.45
G to V	1.04	1.02, 1.06	-7.49	-13.28, -1.24
V to H	0.95	0.93, 0.97	-8.37	-11.05, -5.64
Beyond H	1.91	1.79, 2.03	+101.03	+88.67, +114.64

Supplemental Table S3: Linear regression analysis between the log_{10}-transformed per capita cumulative case count and log_{10}-transformed population size using cumulative incidence for sixteen regions.

**Time point**	**Slope**	**95% confidence interval**	**P value**
June 30, 2020	1.59	0.91, 2.28	0.0002
August 31, 2020	1.17	0.53, 1.82	0.0016
October 31, 2020	1.19	0.54, 1.84	0.0014
December 31, 2020	1.21	0.57, 1.85	0.0011
February 28, 2021	1.10	0.62, 1.58	0.000247
April 30, 2021	0.94	0.53, 1.35	0.000216
June 30 2021	0.94	0.52, 1.35	0.00024
August 31 2021	0.91	0.48, 1.34	0.00050
October 31 2021	0.86	0.45, 1.28	0.00052
December 31 2021	0.90	0.47, 1.33	0.00048

Supplemental Table S4: Time-lag correlation coefficients between the 3-day moving average of daily number of new COVID-19 cases, and 7-day moving average of relative mobility (percentage change from baseline) for trips to retails and recreation, grocery and pharmacy, workplace, and residential categories over the first 90 days of the pandemic in Ghana using the assumed date of infection.[b]

**Lag (days)**	**Retails and recreation**		**Grocery and pharmacy**		**Workplace**		**Residential**	
	**r**	**P value**	**r**	**P value**	**r**	**P value**	**r**	**P value**
**-3**	0.042	0.693	0.172	0.103	0.118	0.263	0.067[a]	0.528
**-2**	0.069	0.516	0.205	0.052	0.149	0.159	0.044	0.629
**-1**	0.096	0.363	0.237	0.024	0.178	0.091	0.022	0.836
**0**	0.123	0.242	0.271	0.010*	0.209	0.047*	0	0.999
**1**	0.167	0.113	0.307	0.004*	0.248	0.019*	-0.042	0.687
**2**	0.208	0.048*	0.341	0.001*	0.291	0.006*	-0.085	0.418
**3**	0.243[a]	0.021*	0.366[a]	0.001*	0.328[a]	0.002*	-0.124	0.240

*p<0.05

[a] The model with the largest (absolute value) correlation coefficient in each category.

[b] Data for the first 90 days was used to observe the impact of relaxation of social restrictions on mobility changes.

Supplemental Table S5: Time-lag correlation coefficients between median 7-day sliding window R_t of COVID-19 and 7-day moving average of relative mobility (percentage change from baseline) for trips to retails and recreation, grocery and pharmacy, workplace, and residential categories over the first 90 days of the pandemic in Ghana using the assumed date of infection.[b]

**Lag (days)**	**Retails and recreation**		**Grocery and pharmacy**		**Workplace**		**Residential**	
	**r**	**P value**	**r**	**P value**	**r**	**P value**	**r**	**P value**
**-3**	0.204[a]	0.053	0.061[a]	0.562	0.137	0.192	-0.295	0.005*
**-2**	0.203	0.054	0.055	0.602	0.139[a]	0.188	-0.310	0.003*
**-1**	0.197	0.062	0.042	0.691	0.130	0.216	-0.317	0.003*
**0**	0.197	0.061	0.031	0.766	0.124	0.240	-0.328[a]	0.002*
**1**	0.164	0.119	0.004	0.972	0.099	0.346	-0.297	0.005*
**2**	0.133	0.207	-0.020	0.851	0.072	0.497	-0.264	0.012*
**3**	0.108	0.304	-0.033	0.755	0.051	0.631	-0.238	0.024*

*$p<0.05$ [a] The model with the largest (absolute value) correlation coefficient in each category.

[b] Data for the first 90 days was used to observe the impact of relaxation of social restrictions on mobility changes.

Supplemental Figure Captions and Legends:

Supplemental Figure S1: Cumulative case count as of December 31, 2021 in Ghana by region.

Supplemental Figure S2: Cumulative case count per 10,000 population as of December 31, 2021 in Ghana by region.

Supplemental Figure S3: The daily number of new cases (left panel), 7-day sliding window R_t (middle panel) and nonoverlapping window R_t (right panel) estimated using the Cori et al. method in the 'EpiEstim' package, in the Central, Eastern, Volta and Western regions, March 12, 2020—December 31, 2021. The government policies represented by the alphabets in the figure are: A = restrictions of all travels to Ghana, suspension of social gatherings, school closure, mandatory 14-day quarantine for all travelers; B = closure of all borders to human traffics; C = mandatory wearing of facemasks at all businesses and organizations; D = relaxation of restrictions at social gatherings; E = deployment of personnel to monitor COVID-19 cases in high schools; F = reopening of international borders; G= reopening of schools, V = vaccination rollout, H = Christmas festivities in 2021.

Supplemental Figure S4: Results from the Generalized Growth Model. Upper panel shows the estimated reproduction number of 1.8 (95% CI: 1.7, 2) for Ghana with 95% CI from March 12, 2020, to March 26, 2020. The growth rate, *r*, 0.84 (95% CI: 0.61, 1.1), deceleration growth parameter, *p*, 0.8 (95% CI: 0.74, 0.87). The lower panel shows the GGM fit of the daily reported number of new infections using the first 15 days of data.

Supplemental Figure S5: Google mobility trends by type of mobility: retail and recreation (first panel), grocery and pharmacy (second panel), workplaces (third panel) and residential (fourth panel) from February 15, 2020, to December 31, 2021. Both the original data (bar) and the 7-day moving average of mobility data (red line) are displayed.

Supplemental Figure S6: COVID-19 testing positivity rate in Ghana by date of report from May 5, 2020 to October 30, 2021.

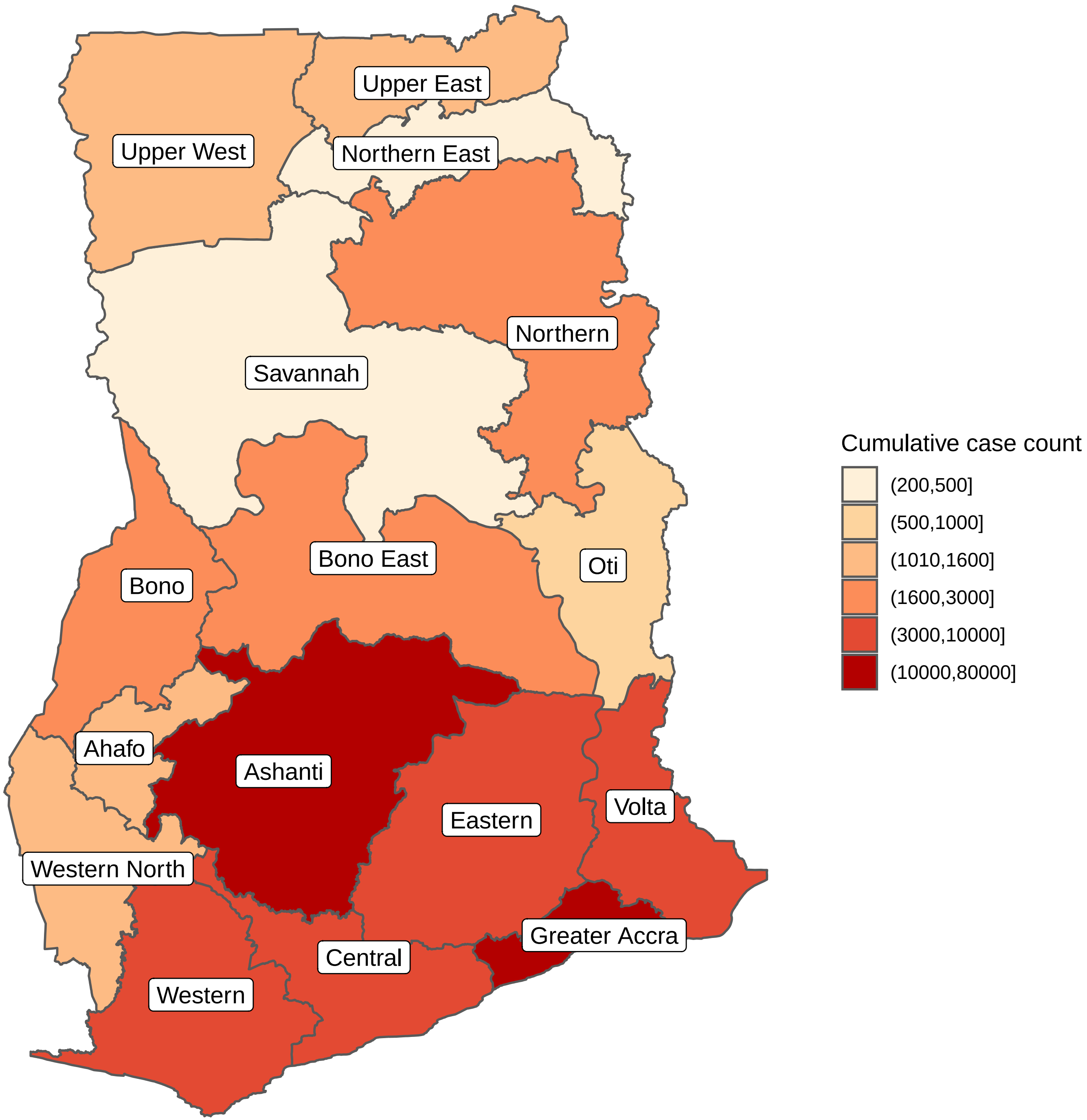

Upper East
Upper West
Northern East
Northern
Savannah
Oti
Bono East
Bono
Ahafo
Ashanti
Eastern
Volta
Western North
Greater Accra
Central
Western
Cumulative case count
(200,500]
(500,1000]
(1010,1600]
(1600,3000]
(3000,10000]
(10000,80000]

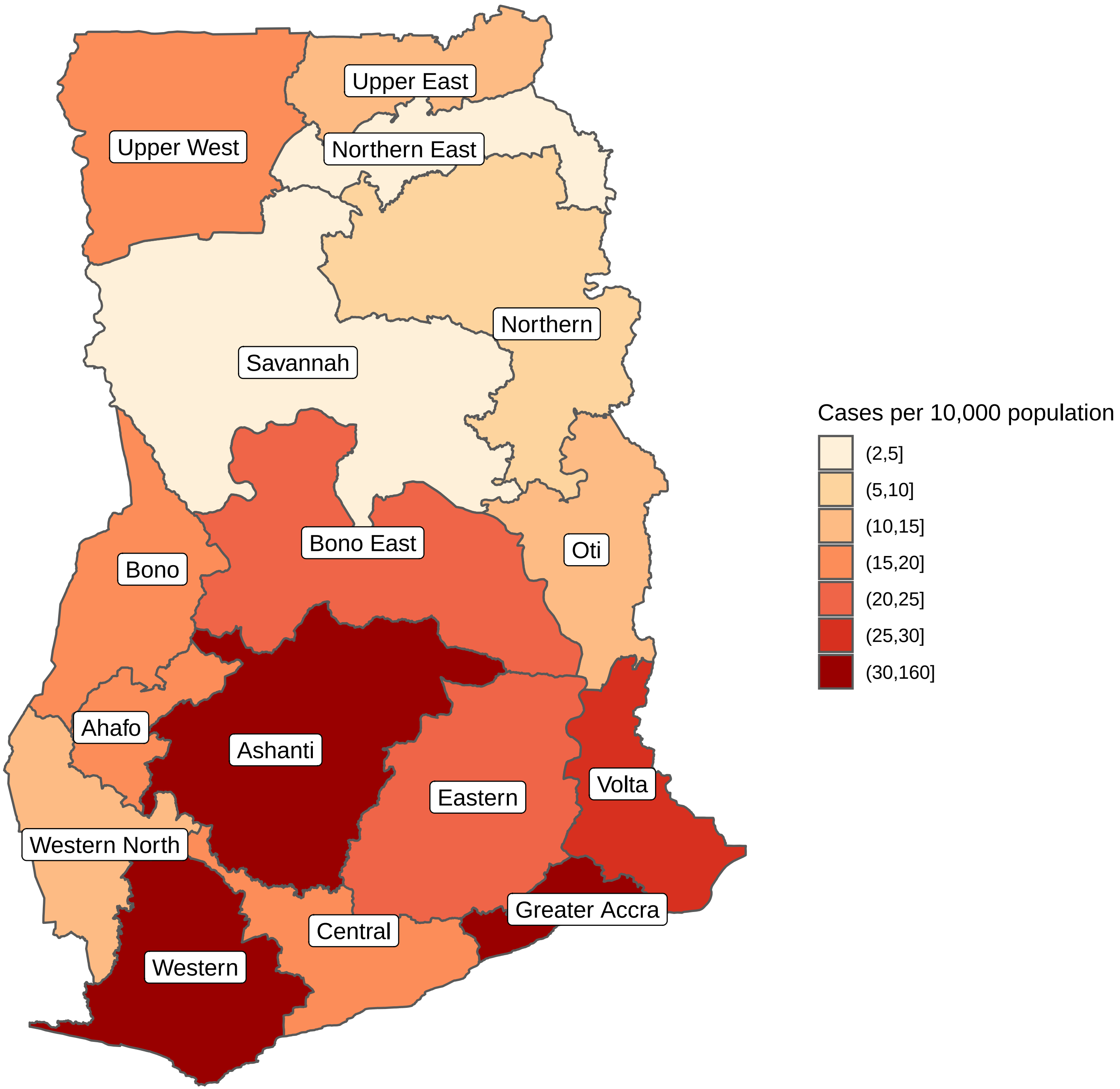
Upper East
Upper West
Northern East
Northern
Savannah
Bono East
Oti
Bono
Ahafo
Ashanti
Volta
Eastern
Western North
Greater Accra
Central
Western
Cases per 10,000 population
(2,5]
(5,10]
(10,15]
(15,20]
(20,25]
(25,30]
(30,160]

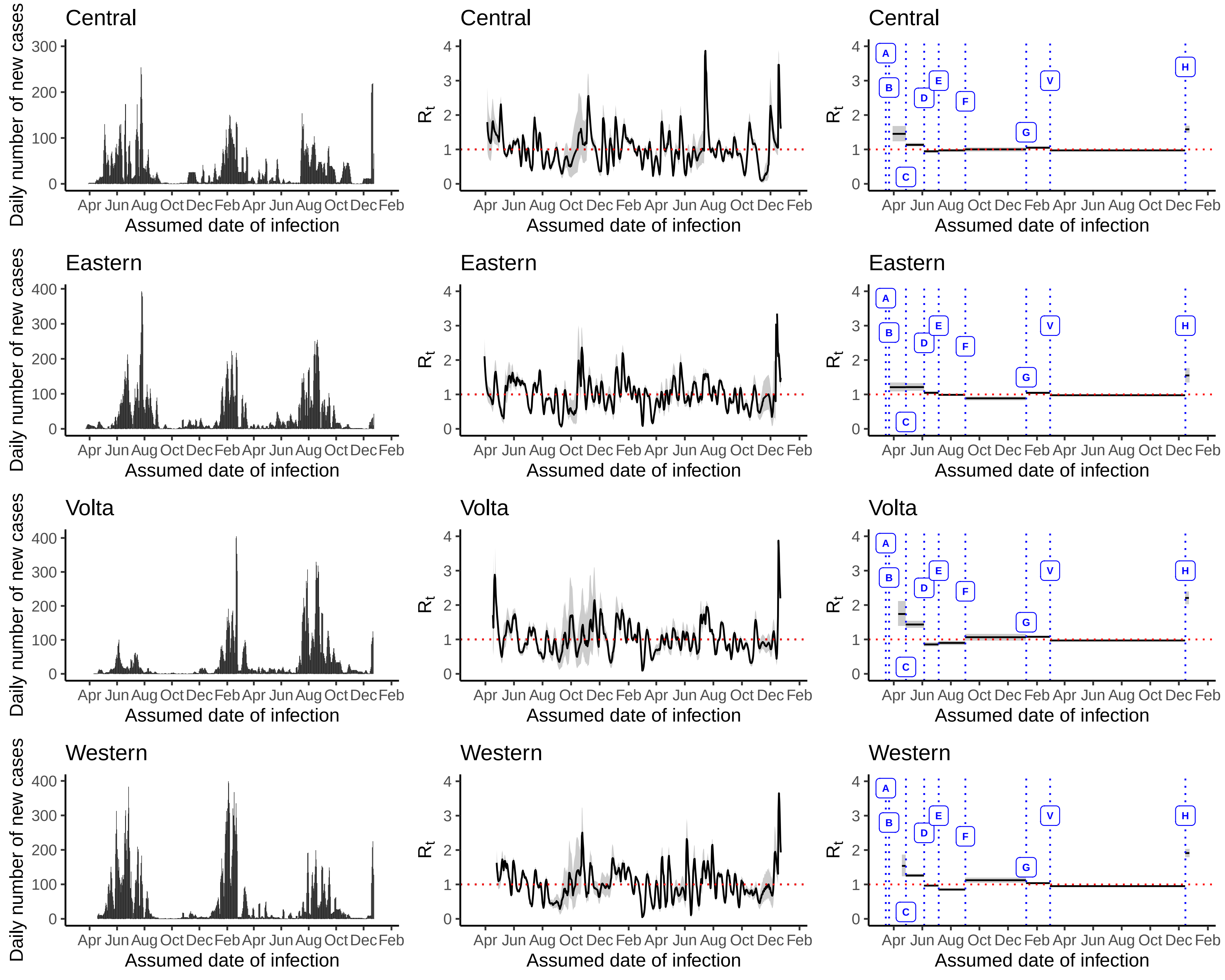
Central
Eastern
Volta
Western
Daily number of new cases
Rt
Assumed date of infection
Apr Jun Aug Oct Dec Feb Apr Jun Aug Oct Dec Feb
A
B
C
D
E
F
G
V
H

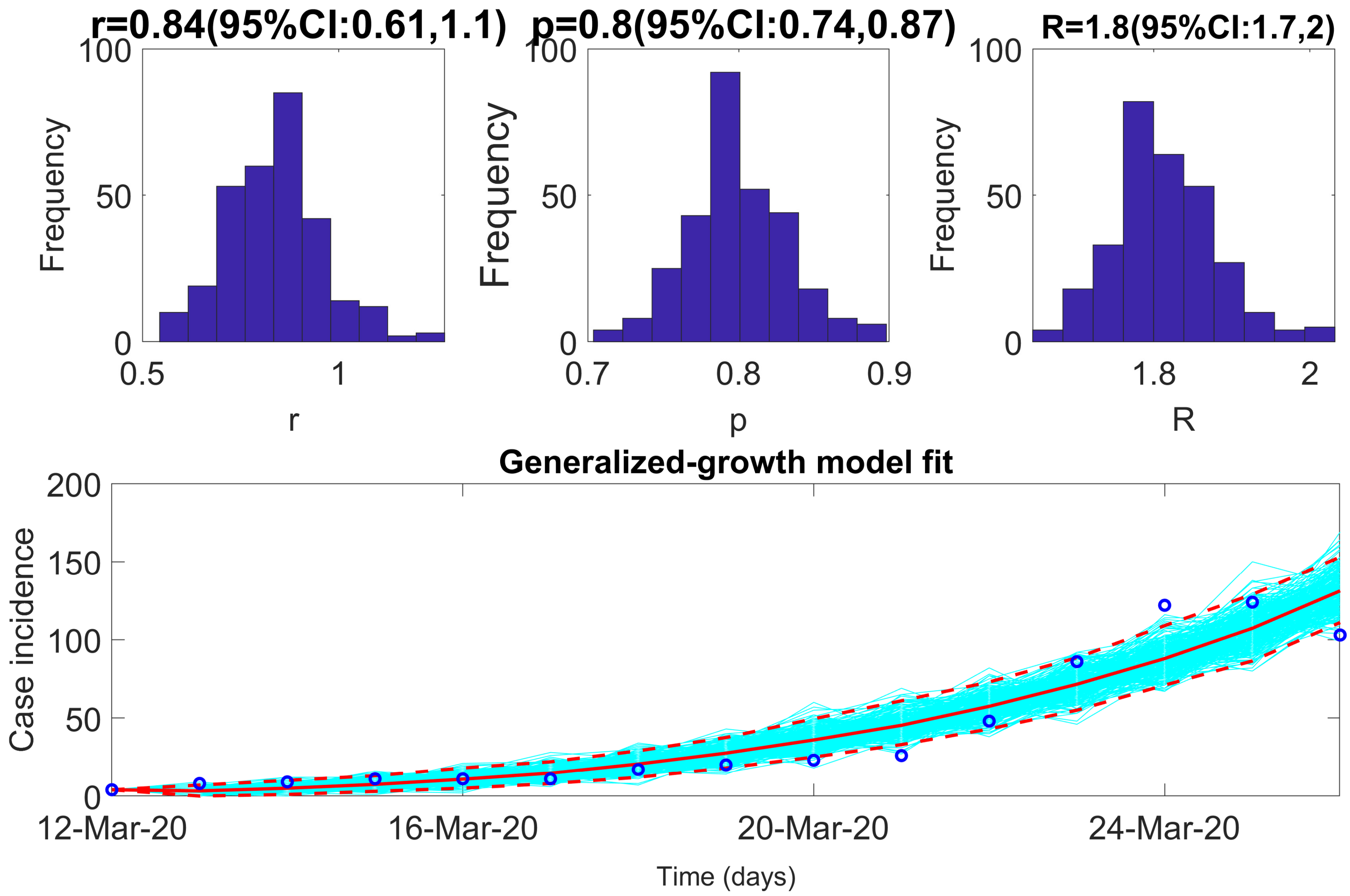

r=0.84(95%CI:0.61,1.1)
p=0.8(95%CI:0.74,0.87)
R=1.8(95%CI:1.7,2)
Frequency
100
50
0
0.5
1
r
0.7
0.8
0.9
p
1.8
2
R
Generalized-growth model fit
Case incidence
200
150
100
50
0
12-Mar-20
16-Mar-20
20-Mar-20
24-Mar-20
Time (days)

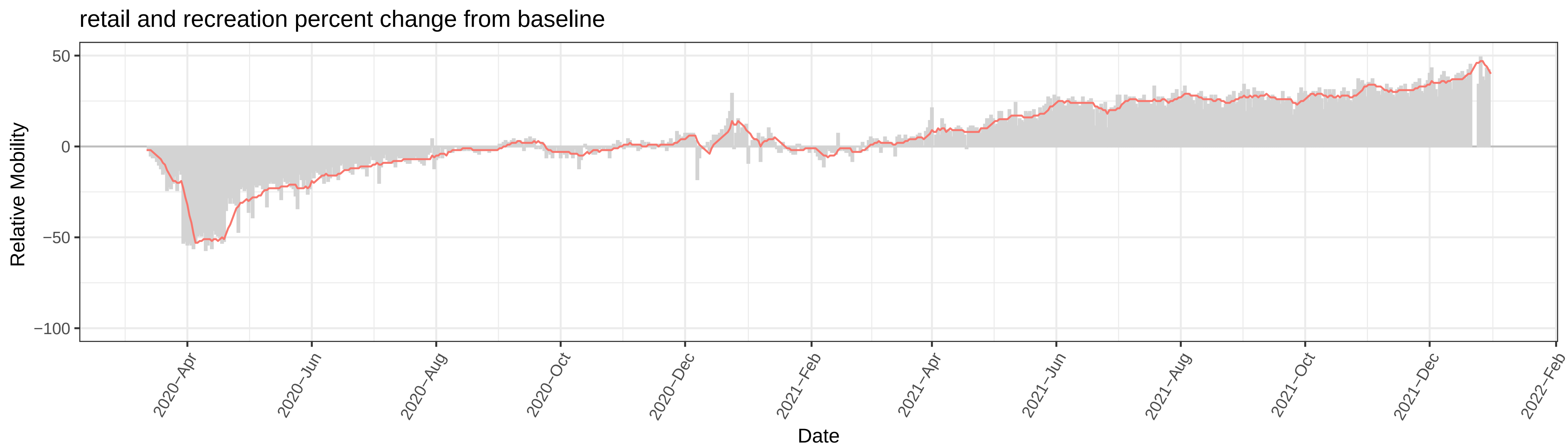
retail and recreation percent change from baseline
Relative Mobility
50
0
−50
−100
2020−Apr
2020−Jun
2020−Aug
2020−Oct
2020−Dec
2021−Feb
2021−Apr
2021−Jun
2021−Aug
2021−Oct
2021−Dec
2022−Feb
Date

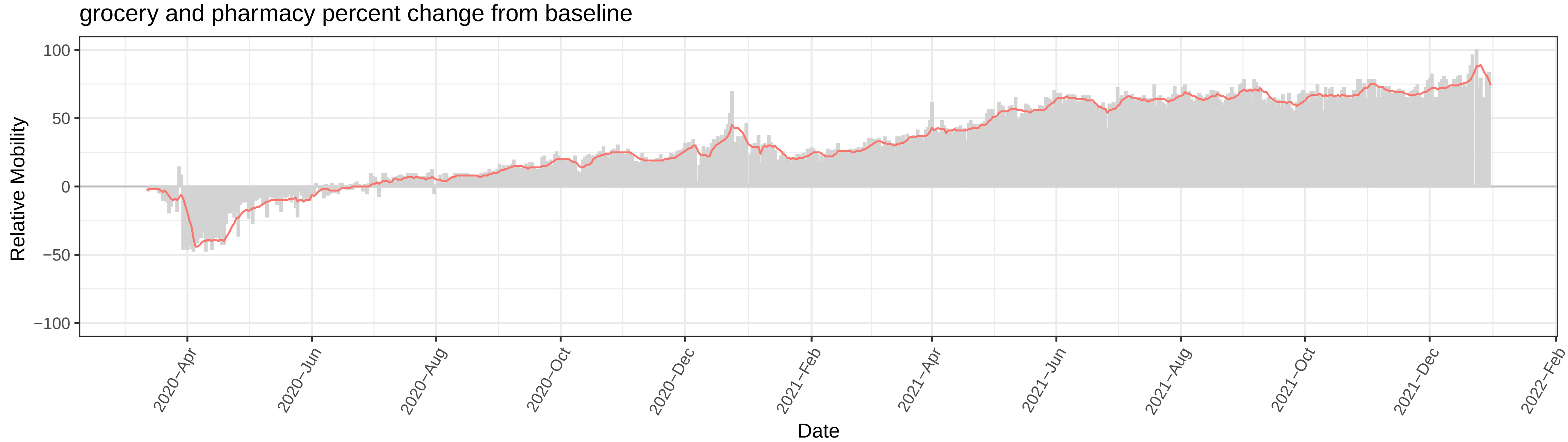
grocery and pharmacy percent change from baseline
Relative Mobility
100
50
0
−50
−100
2020−Apr
2020−Jun
2020−Aug
2020−Oct
2020−Dec
2021−Feb
2021−Apr
2021−Jun
2021−Aug
2021−Oct
2021−Dec
2022−Feb
Date

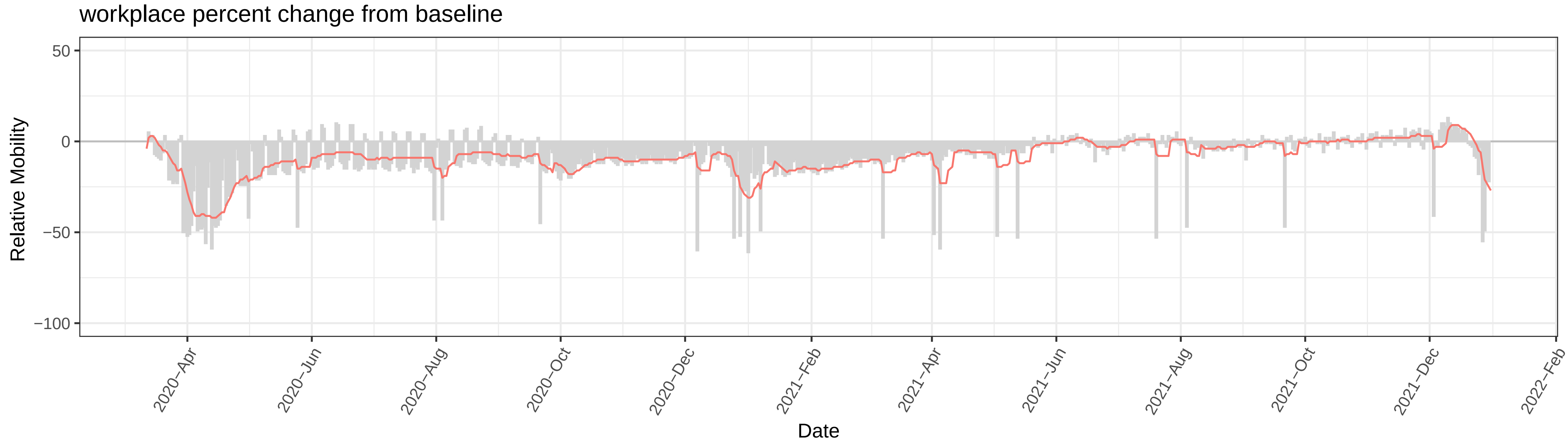
workplace percent change from baseline
Relative Mobility
50
0
−50
−100
2020−Apr
2020−Jun
2020−Aug
2020−Oct
2020−Dec
2021−Feb
2021−Apr
2021−Jun
2021−Aug
2021−Oct
2021−Dec
2022−Feb
Date

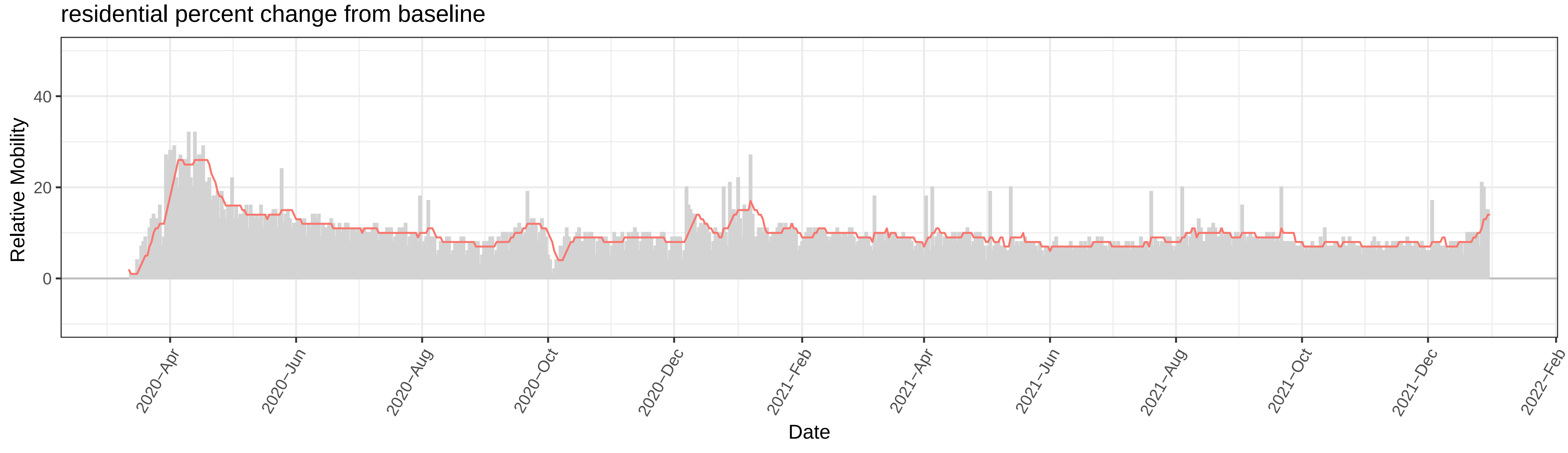
residential percent change from baseline
Relative Mobility
40
20
0
2020−Apr
2020−Jun
2020−Aug
2020−Oct
2020−Dec
2021−Feb
2021−Apr
2021−Jun
2021−Aug
2021−Oct
2021−Dec
2022−Feb
Date

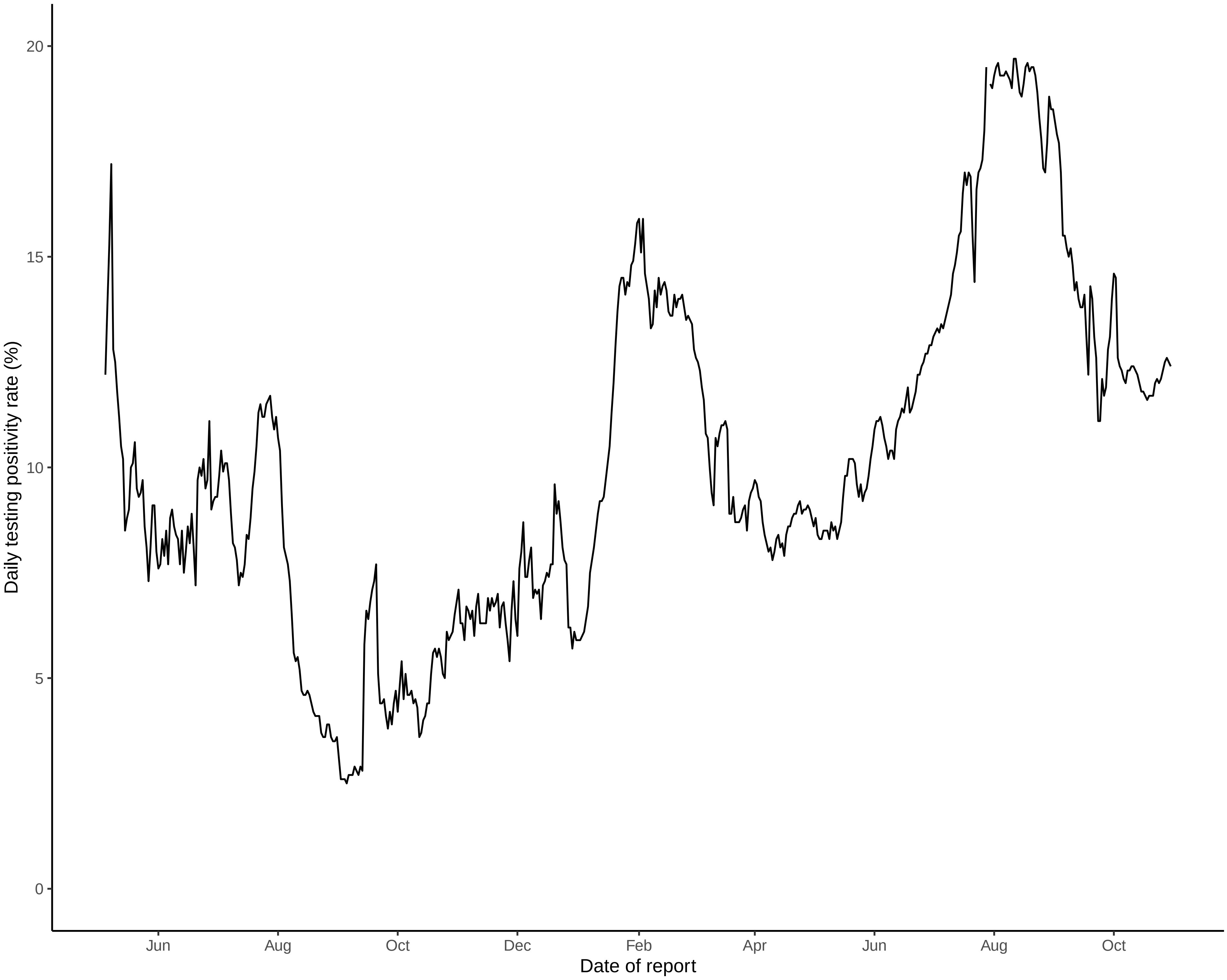

20
15
10
5
0
Daily testing positivity rate (%)
Jun
Aug
Oct
Dec
Feb
Apr
Jun
Aug
Oct
Date of report
